# On Ice-Water in the Treatment of Croup

**Published:** 1861-02

**Authors:** J. A. McFarland

**Affiliations:** Tiffin


### ON ICE-WATER IN THE TREATMENT OF CROUP.

BY J. A. McFARLAND, M. D., TIFFIN.

It will scarcely be necessary to offer any apology, unless on account of tardiness, for inviting the attention of the profession to the use of *Ice-water*, in the treatment of croup.

Fifteen years ago I prepared an article for one of the medical journals on this subject; but seeing, about that time, in one of the newspapers, something very much like "my thunder," I concluded to remain silent. Believing, however, that very few regular practitioners have made any trials of this remedy, I propose, very briefly, to return to the subject, with the hope of, at least, eliciting some profitable discussion.

It was in January, 1843, more than seventeen years since, that I first applied cold applications, in the management of croup. During the treatment of a most violent attack, after the ordinary means had been tried without any apparent benefit, and when fairly puzzled what next to do for the relief of the little sufferer, it occurred to my mind, on placing my hand over the child's throat, that the leading indication was to subdue the burning heat, the result of local inflammatory action; and that nothing could be better for that purpose than ice-water applied directly over the inflamed parts. This was immediately done; and in ten or fifteen minutes a most wonderful improvement was evident. Never have I witnessed, in my whole professional experience, a more rapid and delightful change, than in this my first trial of ice-water in croup.

It was but natural that so gratifying a result would lead to further trials. Up to this time, in my own practice, they must have been repeated at least two hundred times. To this may be added, that no time was lost in communicating the history of my first case to my professional brethren of this city, all of whom, I believe, are in the habit of resorting, very confidently, to the use of cold water in all croupal affections. For my own part, I can truly say, if I were confined to a single article in the treatment of tracheitis, I would prefer cold water to all others. By its judicious employment, commencing before the formation of false membrane, there will be little or no danger of a fatal termination. Even when diphtheritic formations have commenced, there is no surer way of arresting their progress.

It is neither necessary nor proper, however, to confine ourselves exclusively to a single remedy. Whilst, in severe cases, our reliance is mainly on ice-water, recourse is had, as occasion requires, to ipecac, and alum, as emetics; enemata, castor oil, or calomel to evacuate the bowels; the warm bath, anodynes, and solution of nitras argenti.

I must here enter my solemn protest against the frequent repetition of antimonial emetics. They have doubtless been the cause of many a death among young children. Even a single emetic of tartarized antimony, or its long continued use as a nauseant, in delicate infants, with an irritable condition of the alimentary canal, can never be regarded as safe or judicious; and it may be laid down as a good rule to eschew and discourage all such practice. Ipecac, and alum, either singly or combined, will be found equally efficacious, and infinitely safer.

In applying the remedy under consideration, we use folds of muslin or linen, large enough to cover the whole throat and upper part of the sternum. The wet cloth, wrung just enough to prevent dripping, ought to be well covered with several thicknesses of dry flannel, and then both should be secured with a small handkerchief.

When we wish to have the applications very cold, it is best to have two wet cloths, using them alternately, as fast as they become warm. This course should be continued till the disease is thoroughly subdued. When treatment is commenced early, a few hours may suffice; in neglected cases, several days are sometimes required.

In some cases, water fresh from the well or cistern will be sufficiently cold; but as a general rule, the preference should be given to ice-water.—*Columbus Review.*

